# Workplace bullying of psychiatric trainees: systematic review

**DOI:** 10.1192/bjb.2024.58

**Published:** 2025-10

**Authors:** Paul A. Maguire, Fiona A. Wilkes, Stephen Allison, Tarun Bastiampillai, Matt Brazel, Jeffrey C. L. Looi

**Affiliations:** 1 The Australian National University School of Medicine and Psychology, Canberra, Australia; 2 Consortium of Australian Academic Psychiatrists for Independent Policy and Research Analysis (CAPIPRA), Canberra, Australia; 3 Flinders University, Adelaide, Australia; 4 Monash University, Melbourne, Australia

**Keywords:** Bullying, workplace, psychiatric trainee, perpetrator, reporting

## Abstract

**Aims and method:**

We aimed to systematically review primary studies exploring workplace bullying of psychiatric trainees, including rates, forms of bullying, perpetrators and help-seeking. We searched Ovid MEDLINE, PubMed, CINAHL, PsycINFO and Embase using PRISMA guidelines. The inclusion criterion was primary research papers surveying or interviewing psychiatry trainees with respect to perceived workplace bullying by staff members. Exclusion criteria were secondary research papers and papers whose only focus was bullying by patients or carers.

**Results:**

Substantial levels of bullying were reported in all five included studies. Perpetrators were often reported to be consultants, managers or peers. Most trainees did not obtain help for bullying and harassment. All of the studies had methodological limitations.

**Clinical implications:**

Concerning levels of workplace bullying have been reported by psychiatric trainees in the UK and abroad. Further methodologically robust studies are required to evaluate the current levels and nature of this bullying, and strategies to prevent and manage it.

Trust, safety and respect are essential for psychiatric trainees to learn the knowledge and skills they need to provide effective treatment for their patients. Bullying may disrupt this learning and patient care, as well as cause distress and depressive and anxiety symptoms in trainees.^1^ Risks of workplace bullying are higher for trainees working in large hierarchical organisations such as hospitals. The provision of a safe and respectful learning environment within health systems is often the immediate responsibility of consultant psychiatrists to whom the trainee is apprenticed. Thus, the prevention of workplace bullying of trainees is a shared and collective responsibility of psychiatrists and health systems.

There is no universally accepted definition of workplace bullying, but most conceptualisations of bullying comprise three key components: a power imbalance; a negative and unfavourable impact on the recipient; the bullying behaviour is recurrent. A useful definition of bullying is provided by the Royal College of Psychiatrists: ‘Bullying at work is an abuse of power or position. It is offensive discrimination through persistent, vindictive, cruel or humiliating attempts to undermine, criticise, condemn, hurt or humiliate either an individual or a group of employees’.^2^ Bullying can take many forms and may be placed on a spectrum ranging from incivility, unjustified criticism, demeaning innuendo, sarcasm and exclusion through to sexual harassment, intimidation and frank physical violence.^3,4^ Workplace legislative definitions emphasise a risk of bullying behaviour to occupational health and safety. For instance, the Fair Work Act 2009 in Australia defines bullying as occurring when ‘an individual or group of individuals repeatedly behaves unreasonably towards a worker or groups of workers at work, and the behaviour creates a risk to health and safety’.^5^ Some authors place bullying under the broad umbrella of ‘counterproductive workplace behaviours’ (CWBs), a term that includes all ‘harmful behaviours at work’, with a subcategory of aggression, where bullying belongs.^6^ However, some forms of bullying, although inherently aggressive in nature, can be very subtle, including: staring or avoiding eye contact; not returning communications; gossip; ignoring; isolating and exclusion.^4^

Recent estimations are that workplace bullying affects hundreds of millions of people each year, with substantial prevalence rates around the world. Reported rates vary across countries, between public and private sectors, and between genders. In a nationwide survey of 70 organisations in the UK, 10.6% of respondents reported being victims of workplace bullying.^7^ Within the UK public sector this was even higher, at 34%.^8^ The prevalence of workplace bullying in New Zealand and Australia has been found to be 18% and 25–50% respectively.^9–11^

Unfortunately, junior doctors frequently experience bullying in the workplace. A UK study found that 84% of junior doctors (ranging from house officers through to senior registrars) reported at least one incident of bullying in their work lives, with 37% of the doctors surveyed reporting that they had been bullied during the previous year.^12^ Junior doctors training in psychiatry, both in the UK and abroad, are not exempt from workplace bullying.^13–17^ However, research on workplace bullying of psychiatric trainees is a much-neglected area and we were unable to find any previous systematic reviews. Therefore, we propose that this review be used as a clear building block on which further research can be undertaken.

In this systematic review our aim is to evaluate primary studies exploring bullying of psychiatric trainees in their workplaces. Although trainees may experience negative interactions with patients or carers, this is not generally regarded as workplace bullying, so we have focused on bullying by staff members. The specific aims of this systematic review are to address the following research questions: (a) What are the rates of bullying of psychiatric trainees in the UK and abroad? (b) What is the nature and form of bullying incidents of psychiatric trainees? (c) Who are the perpetrators of bullying of psychiatric trainees? (d) What steps do psychiatric trainees take to report a bullying incident and seek help?

## Method

### Protocol and registration

We registered our systematic review with Prospero on 30 August 2023 (CRD42023455231) (https://www.crd.york.ac.uk/PROSPERO/).

### Databases and search strategy

**Fig. 1:**
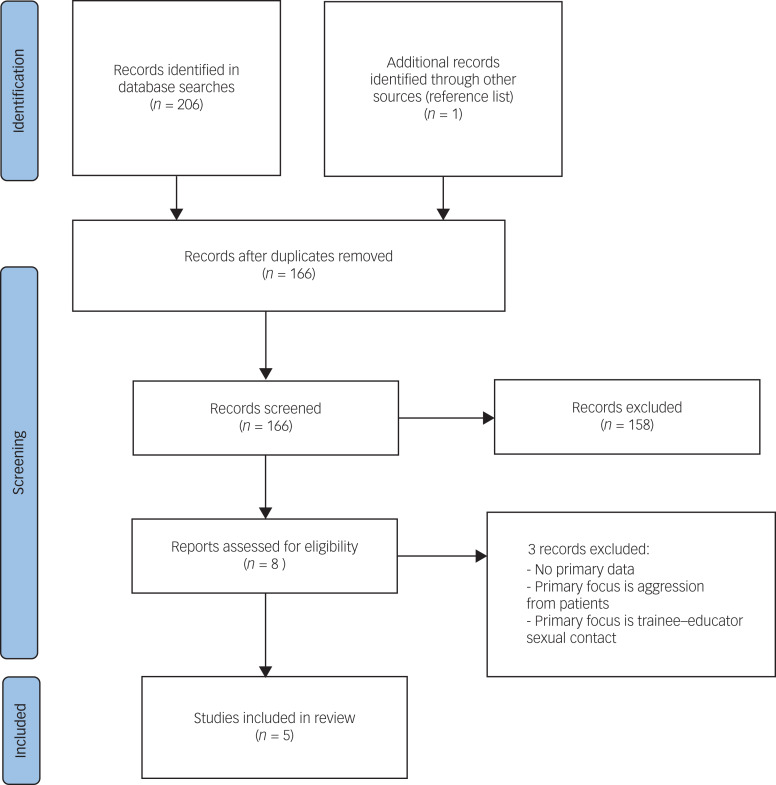
PRISMA flow diagram.

Preferred Reporting Items for Systematic Reviews and Meta-Analyses (PRISMA) guidelines were followed when conducting this systematic review (Fig. 1). A comprehensive search was performed using the databases Ovid MEDLINE, PubMed, CINAHL, PsycINFO, and Embase, from 1 January 1980 to 1 September 2023. Search terms included: (Bullying OR harassment OR intimidation OR discrimination OR workplace abuse OR abuse in the workplace) AND (psychiatr* trainee* OR psychiatr* registrar* OR psychiatr* resident* OR psychiatr* intern* OR specialist registrar* OR trainee psychiatrist*), where * represents plural forms of the relevant word or different characters of the end of the relevant word, such as psychiatry, psychiatric. Search strategies for each database can be found in the Supplementary Appendix available at https://doi.org/10.1192/bjb.2024.58. The reference lists were manually searched to identify any further relevant articles.

### Eligibility criteria

The inclusion criterion was: primary research papers surveying or interviewing psychiatry trainees with respect to perceived bullying (including harassment, intimidation or discrimination) by staff, including, but not limited to, supervisors and peers.

Exclusion criteria were: (a) secondary research papers commenting on primary research or papers providing reflection, speculation or commentary with no new data; (b) papers whose only focus was bullying of psychiatric trainees by patients or carers.

### Study selection

The titles and abstracts from the search were reviewed independently by two authors (P.A.M. and J.C.L.L.) to determine whether or not they met the eligibility criteria. There was full consensus between these authors and therefore a third author was not required to resolve a disagreement.

### Data extraction

Relevant study data were extracted (14 September 2023) from identified papers by one author (P.A.M.) and confirmed by a second author (J.C.L.L.). This information included: author(s), year, country, study design, participant numbers and characteristics (if available), nature of bullying incidents, instruments/outcome measures used, rates of bullying, perpetrators of bullying, reporting the bullying incident(s) and seeking help.

## Results

As shown in the PRISMA flowchart (Fig. 1), 206 articles were identified with our search strategy and 1 additional study was found manually from the reference lists of these articles. In total, 41 duplicates were removed and a further 161 articles were excluded as they did not meet the eligibility criteria. This left five articles, all of which were cross-sectional surveys in the form of self-report questionnaires (Table 1). Two studies were surveys that used the Quine bullying questionnaire^1^ (Box 1) and a five-option question on perpetrators of the bullying (Pakistan, UK).^13,16^ Two studies were surveys that used local purpose-designed, non-validated questionnaires consisting of open-ended questions (both UK)^14,15^ and one used a partially validated instrument examining trainee's experiences more generally but with a section on adverse experiences (Australia).^17^ Since there was a lack of common bullying assessment instruments, and considerable heterogeneity in study design and sociocultural context (UK, Pakistan, Australia), it was not feasible to perform a meta-analysis.

**Table 1:** Summary of included studies

Authors/Year	Country	Study design	Participants	Instruments/Outcome measures	Findings
Ahmer et al (2009)^16^	Pakistan	Cross-sectional survey (questionnaire)	60 psychiatric trainees (47 male, 11 female, 2 did not identify their gender) working in four different regions in Pakistan (Punjab, Sindh, North West Frontier Province (NWFP) and Baluchistan); 71.4% response rate (from 84 trainees)	Quine bullying questionnaire (experience of any of 21 bullying behaviours in previous 12 months); 5-option question on perpetrator(s) of bullying (consultant, nurse, manager, patient or peer)	80% of respondents reported ≥1 bullying behaviour(s) in preceding 12 months (no differences between demographic variables: male *v.* female; single *v.* married; urban *v.* rural background; different work provinces) Most common bullying behaviours: persistent attempts to belittle and undermine work (41.7%) and persistent attempts to humiliate in front of colleagues (41.7%). Most commonly reported perpetrators: consultants (73.3%); peers (35.6%); managers (22.2%); patients (15.6%); and nurses (13.3%)
Hoosen & Callaghan (2004)^13^	England	Cross-sectional survey (questionnaire)	177 psychiatric trainees (93 male, 84 female) working in the West Midlands; 76% response rate (from 232 trainees)	Quine bullying questionnaire (experience of any of 21 bullying behaviours in previous 12 months); 3-option question (Yes/No/Unsure): ‘Do you know where to obtain help or whom to contact if you are bullied’; it is not clear from the methods how perpetrators were assessed	47% of respondents reported ≥1 bullying behaviour(s) in preceding 12 months (no difference between demographic variables: male *v.* female; White *v.* Asian; local *v.* foreign). 46% reported knowing who to contact in the event of being bullied. Reported bullying perpetrators were: non-medical staff (28%); senior medical staff (27%); patients (20%); managers (16%); and peers (9%)
Reddy & Kaplan (2013)^15^	England	Cross-sectional survey (self-report questionnaire)	30 psychiatric trainees (17 male, 13 female) in the Northern Deanery training schemes (Durham Trees Valley, East Cumbria, Northumbria and West Lakes); 61% response rate (from 49 trainees)	A (non-validated) questionnaire consisting of open-ended questions was used to explore experiences of verbal and/or physical abuse in the workplace. Questions about the following were included: frequency of abuse; where the abuse occurred; whether was help sought and who from; who was abusive; whether the abuse was related to gender, ethnicity, religion, accent, disability or other; whether participant had received formal teaching on how to deal with abuse	77% reported abuse (all of these included verbal abuse, 9% reported physical abuse as well). Reported abuse perpetrators were patients (75%), carers (21%) and a consultant (4%, 1 trainee's experience). Perceived reasons by trainees for abuse included: being part of the job; gender; ethnicity; and accent. 52% of trainees (12) reported seeking and receiving support: 33% (4) from nursing staff; 25% (3) from a peer; 25% (3) from the educational supervisor; 8% (1) from a consultant; and 8% (1) from a medical secretary
Morgan & Porter (1999)^14^	UK	Cross-sectional survey (self-report questionnaire)	85 psychiatric trainees (37 male, 48 female) in a UK ‘large psychiatric rotation’; 85% response rate (from 100 trainees)	Purpose-designed (non-validated) questionnaire exploring experiences of and attitudes towards unwanted sexual contact at work, including: nature of sexual harassment; perpetrator's status (staff *v.* patient); impact of patient's diagnosis; and how much of a problem sexual harassment is for the profession	64% reported unwanted sexual contact from staff: uninvited sexual teasing, jokes, remarks, questions, looks or gestures (49%); uninvited and deliberate touching, leaning over or cornering (23%); uninvited pressure for dates (16%); and uninvited letters, telephone calls or material of a sexual nature (8%)
Kozlowska et al (1997)^17^	Australia	Cross-sectional survey (self-report questionnaire)	110 psychiatric trainees with >I year of training; 82.6% response rate (from 132 trainees) but 3 respondents were excluded (1 had <1 year of training, and 2 returned uncompleted questionnaires)	Training Impact Questionnaire (Adverse Experiences Section V)	41.5% reported severe criticism or humiliation (of a trainee or a fellow trainee) by a consultant; 23% reported malicious accusations against them; 13% reported sexual harassment by a staff member or colleague; 3% reported physical intimidation by a staff member

### Rates of bullying

Substantial levels of bullying were reported in all of the studies. The two surveys using the Quine bullying questionnaire found that, respectively, 80 and 47% of respondents reported at least one bullying event over the preceding 12 months.^13,16^ Only one of these studies quantified the number of respondents indicating bullying experiences for each of the 21 items in the questionnaire.^16^ In that study, physical violence as a form of bullying was relatively low (5%), whereas bullying events forming a threat to the trainee's professional status (belittling and undermining the trainee's work and attempting to humiliate them) were high (41.7%).^16^

In the remaining three studies, the research definitions of bullying were narrower.^14,15,18^ In a study focusing specifically on unwanted sexual contact (as the form of bullying) 64% of trainees reported bullying.^14^ In a study exploring verbal and/or physical abuse 77% of trainees reported that they had been verbally abused, with 9% reporting physical abuse as well.^15^ The final study (published in two separate parts) used an instrument examining respondents’ training experiences across a range of domains.^17,19^ A sub-section of this instrument focused on adverse experiences. Within this section, there were three questions that could be considered to explore bullying: 41.5% reported severe criticism or humiliation (of themselves or a fellow trainee) by a consultant, 23% reported malicious accusations against them and 13% reported sexual harassment by a staff member or colleague.

The studies in this systematic review did not find statistically significant differences in overall reported bullying between male and female psychiatric trainees.

### Forms of bullying

Trainees may experience a wide range of events as bullying. The 21-item questionnaire developed by Quine and employed in two studies^13,16^ classifies bullying into six categories: threat to professional status (e.g. persistent attempts to belittle and undermine the trainee's work); threat to personal standing (e.g. persistent attempts to demoralise the trainee); isolation (e.g. freezing out, ignoring or excluding); overwork (undue pressure to produce work); destabilisation (withholding necessary information from the trainee); and discrimination on the basis of race or gender (Box 1).^12^ It does not specifically include sexual harassment but does encompass verbal and non-verbal threats and physical violence, as well as a broad range of psychological and organisational items.

Box 1 Quine bullying questionnaire items^11^

1. Persistent attempts to belittle and undermine your work
2. Persistent unjustified criticism and monitoring of your work
3. Persistent attempts to humiliate you in front of colleagues
4. Intimidatory use of discipline/competence procedures
5. Undermining your personal integrity
6. Destructive innuendo and sarcasm
7. Verbal and non-verbal threats
8. Making inappropriate jokes about you
9. Persistent teasing
10. Physical violence
11. Violence to property
12. Withholding necessary information from you
13. Freezing out/ignoring/excluding
14. Unreasonable refusal of applications for leave, training or promotion
15. Undue pressure to produce work
16. Setting of impossible deadlines
17. Shifting goalposts without telling you
18. Constant undervaluing of your efforts
19. Persistent attempts to demoralise you
20. Removal of areas of responsibility without consultation
21. Discrimination on grounds of race or gender

Sexual harassment as a form of bullying was explored in two of the surveys (Table 1).^14,17^ In the Australian study the questionnaire included an item directly enquiring whether there had been sexual harassment by a staff member or a colleague.^17^ The second study explored the occurrence of uninvited sexual behaviours by staff or patients, and whether or not the trainee regarded this behaviour as sexual harassment.^14^ For females, 46% reported experiencing unwanted sexual teasing, jokes, remarks, questions, looks or gestures from colleagues and half of these regarded it as sexual harassment. In contrast, 65% of male trainees experienced these events, but only 21% viewed them as harassment.^14^ Regarding uninvited pressure to go on a date, 23% of female trainees reported experiencing this and 64% of these regarded it as harassment, whereas 11% of male trainees experienced this and none of these regarded it as harassment.^14^

### Perpetrators of bullying

The work role of bullying perpetrators was identified in different ways for each study.

The Pakistan-based study, employing the Quine-developed bullying questionnaire, used a five-option response, nominating specific possibilities (consultant, peers, managers, patients or nurses).^16^ In almost three-quarters of cases (73.3%), the perpetrator was reported to be a consultant. Next, at about half the reported frequency (35.6%), were peers, followed by managers (22.2%), patients (15.6%) and nurses (13.3%).

In contrast, the study of trainees in the West Midlands (England) found that ‘senior medical staff’ accounted for only a little over a quarter (27%) of identified perpetrators, and peers only 9%.^13^ Non-medical staff (not specified further) also accounted for slightly over a quarter of reported bullying perpetrators (28%). Patients were nominated as the perpetrator of bullying behaviour by 20% of respondents, and managers by 16% of respondents.

In the Australian study, information about perpetrators came from specific questions in the survey with a narrower focus on both the type of bullying and the possible perpetrators. These included options of ‘severe criticism or humiliation by a consultant’ (of the trainee himself/herself or observed towards another trainee) reported by 41.5%, and ‘sexual harassment by a staff member or colleague’ reported by 13%.^17^

In the final study, examining UK trainees, questions relating to perpetrators of sexual harassment narrowed the options down to ‘colleagues’ and ‘patients’.^14^ Three-quarters of respondents reported unwanted sexual contact from patients and 64% from staff. The paper appears to use the words ‘staff’ and ‘colleagues’ interchangeably. It is not clear from the text exactly who either comprises.

### Reporting bullying and obtaining help

Three studies collected data on reporting and/or obtaining help for bullying.^13,14^ The UK study of sexual harassment of trainees found that only 25% of respondents who had reported being harassed by staff had informed colleagues about this harassment.^14^

The West Midlands study found that only 46% of respondents reported that they knew who to contact in the event of bullying.^13^ Of the 410 bullying events reported, ‘action was taken’ in 92 instances (just over 22%). However, this reporting was more likely to have a negative outcome for the respondent (61 occasions, 66%) than a positive outcome (31 occasions, 34%). Foreign trainees were less likely to take action compared with local trainees (32 *v.* 60 occasions).

In the Northern Deanery study, exploring verbal and/or physical abuse experienced by 30 psychiatric trainees in training schemes in the north of England, just over a half (52%) of trainees reported seeking support for the bullying incident. The main three sources of help sought were nurses, peers and an educational supervisor, although a consultant and medical secretary were also approached.^15^

## Discussion

There is a concerning level of bullying of psychiatric trainees revealed in the studies in this systematic review. However, caution is required with respect to extrapolating the findings of this review to current psychiatric training contexts, as the number of studies is small and most are not recent.

### What is bullying?

Trainees vary in the way they interpret behaviours of others in the workplace, with different thresholds for identifying a given behaviour as ‘bullying’. Furthermore, there may be a semantic context, whereby some individuals will label unwanted behaviours by staff that are suggestive of racial or gender discrimination as ‘racist or ‘sexist’ respectively, instead of ‘bullying’. Some sexual behaviour, such as uninvited requests for a date, or sexual remarks or looks, are not always viewed as harassment by male trainees.^14^

Although there needs to be a balance in the structure of instruments designed to collect data on bullying and harassment it may be preferable to include a broad range of behaviours under the umbrella of ‘bullying’ rather than having too narrow a focus, and also to enquire whether the trainees viewed those items as bullying or not. The Quine questionnaire is a comprehensive tool for evaluating bullying and harassment behaviour. The earlier (20-item) version did not include, as bullying, discrimination on the basis of race and gender, but these were added in the updated (21-item) version.^1,12^

Qualitative data in the Australian study revealed that several psychiatric trainees viewed unfair criticism and humiliation as just a ‘normal ‘experience in training, rather than a form of bullying.^17^ These experiences included being shouted at in the presence of others, being blamed for a patient suicide and being told that they were incompetent. Yet, ostensibly, these behaviours may be reasonably regarded as bullying in other contexts, and the trainees’ views may reflect either the Australian sociocultural context, or perhaps acclimatisation to prevalent bullying.

There is also the salience of the role of medical practitioners, the training programmes, health system and sociocultural milieu, without which bullying cannot be fully contextualised, and which differ considerably even across the studies included in the review (Pakistan, UK, Australia). For example, although Australia is an Anglophone country, its sociocultural characteristics are considerably different from the UK and from Pakistan, a South Asian country.

### How do bullying rates in psychiatric training compare with those in other medical specialties?

The background levels of bullying in general are concerningly high in healthcare, and a recent international umbrella review found that physicians were the second most commonly affected group (after nurses), at a prevalence of 11.5–78.1%.^20^ Overall prevalence of bullying across the healthcare profession varied by region, with the highest levels in Europe (at 26.4%) and lowest in Southeast Asia (5.3%).^20^ This highlights the need for cultural reference points for bullying rates, even if these are from other medical specialties, to provide some health system and sociocultural context. One might speculate that the apparent disparity in bullying rates between Europe and Southeast Asia may be due, at least in part, to possible underreporting due to differing sociocultural contexts. A cross-sectional survey and interview study of workplace bullying in the UK's National Health Service (NHS) found that 20% of staff (doctors, dentists, nurses, allied health, technicians, administration) reported having been bullied by another staff member, and 43% reported having witnessed bullying, in the preceding 6 months.^21^

The relative rates of bullying experienced by 1852 cardiology trainees in Pakistan was assessed using the Negative Acts Questionnaire-Revised (NAQ-R), with bullying reported by 10.2% of males and 13.4% of females.^22^ The rate of bullying of cardiology physician trainees was 11% in a survey of 1358 respondents in the UK,^23^ with cardiology specialists (80%) and other medical specialists (70%) most commonly implicated in bullying. As a comparator for Australia, the rate of bullying in a surgery survey of 3516 not limited to trainees is very high, at 49.2%,^24^ and most perpetrators were male surgical specialists. Unfortunately, it appears that surgery has very high rates of bullying in the UK and Ireland (60% of 837 trainees) as well.^25^

Australia has a national survey of medical practitioners attached to the medical board registration process, and this reports data as the Medical Training Survey.^26^ In national survey data of Australian psychiatric trainees in 2020–2022, 22% reported personally *experiencing* bullying and harassment while 32% reported *witnessing* bullying and harassment, with similar rates for both of these in 2019–2021.^27^ Australian physician and surgical trainees reported very similar rates, so this may well reflect the overall levels of bullying in the sociocultural milieu of Australian medical training.^27^

### Who are the bullies?

The high proportion (73.3%) of perpetrators reported to be consultants in the Pakistan study may have a cultural context, as suggested by the study's authors.^16^ There is a strongly hierarchical aspect to the health system in Pakistan, with medical practitioners, especially those with a postgraduate qualification, being considered higher in status than nurses and allied health practitioners. There exists an overarching power gradient between consultant supervisors and trainees, and at the time of this Pakistan study (2007), supervisors could disrupt the career trajectory of the trainee by declaring that they were not ready to sit their postgraduate examination, with no appeals permitted in most regions of the country. The substantial level of bullying by peers in the study may possibly reflect the competitive training environment but may possibly also indicate a lack of solidarity among some of the trainee groups.

The lower but still substantial levels of bullying by senior doctors in the UK reported by psychiatric trainees may also have a cultural dimension. As the authors of the West Midlands study suggest, psychiatric training (and medical training more broadly) takes place in UK institutions which have, or have had, a very hierarchical operational structure, and traditionally teaching has employed intimidation and opprobrium, which may promote a culture of bullying and harassment.^13^ The authors point out that there may be cycles of mistreatment whereby those who were bullied go on to bully junior doctors when they themselves become senior clinicians.

Similarly, the reporting of Australian consultants’ demeaning behaviours towards their psychiatric trainees may relate to structural hierarchy.^15^

### Measures of bullying used

There are challenges in standardising and measuring the magnitude and intensity of bullying behaviours. Two of the studies in this review used the Quine bullying questionnaire.^13,16^ This questionnaire was first used by Quine in 1999 when evaluating workplace bullying in an NHS community trust, and subsequently used by Quine and her colleagues to evaluate bullying among junior doctors, doctors undertaking research and postgraduate hospital dentists.^12,28^ Despite a paper in our review stating that the questionnaire has been validated, we could not find any evidence of this.^16^ However, the 20 questions used in the questionnaire (first version) were based on a thorough exploration of the nature, form and contexts of bullying behaviours identified by an extensive literature review, including the six categories formulated by Rayner & Hoel, described above.^29^ The Quine bullying questionnaire simply asks respondents to indicate with a binary yes/no whether they had been persistently subjected to any of the 20 listed behaviours over the preceding 12 months.

A further two studies used their own ‘purpose-built’ questionnaires, which had not undergone evaluation of psychometric properties and therefore may contribute to some weakness and uncertainty in the reliability and validity of the findings.^14,15^

The final study^17,19^ used a partially validated tool (the Training Impact Questionnaire). An initial draft questionnaire was revised after input by five fellow psychiatric trainees. This revised version was endorsed for face validity and comprehensiveness of content coverage by experienced researchers. The authors acknowledged the need for testing for discriminant validity and predictive validity for future research.

Although not used in any of the studies in this review, a widely used tool to assess workplace bullying is the Negative Acts Questionnaire-Revised (NAQ-R).^3^ The NAQ-R has 22 items that assess the occurrence of bullying within the previous 6 months of work as experienced by the respondents. It has been used in the UK and abroad.^3,7^ It has been shown to have sound psychometric reliability and validity. When a sample of 5288 UK employees was analysed the NAQ-R was found to have high internal stability and three underlying factors, consisting of personal bullying, work-related bullying and physically intimidating bullying. However, the NAQ-R was demonstrated also to function as a single factor measure.^3^ Criterion validity was found when an external single item measure of perceived victimisation from bullying correlated highly with the total NAQ-R score as well as the scores on the three factors. Targets of bullying had significantly higher scores on all 22 items compared with non-targets.

### Sequelae of bullying

There were no quantitative data from the review studies relating to sequelae of workplace bullying of psychiatric trainees. However, qualitative data in the Australian study showed reports, by trainees, of reduced self-confidence, distress, fear and feelings of uselessness.^17^ It is unclear whether these consequences were transient or more enduring.

Studies of public health staff, more broadly, provide evidence of important associations with bullying, including reduced job satisfaction, higher stress levels, a greater likelihood of anxiety or depression, more likelihood of reporting wanting to leave work and increased amount of sick leave taken.^1,12,30^ Although these may be a direct result of bullying, there are other possible contexts. Staff who have pre-existing anxiety or depression, or poor coping abilities, may have a lower threshold for labelling behaviours as bullying and be more likely to report them. Alternatively, staff who have pre-existing anxiety or depression may be targeted by bullies. There may also be an increased risk of cardiovascular disease associated with bullying, mediated through comfort eating leading to being overweight.^31^ It should also be borne in mind that bullying behaviours, especially if reported to the medical board, may also do harm to the perpetrator, including loss of employment or de-registration, as well as reputational damage.

### Reporting, seeking help and the path forward

Understanding the barriers to trainees reporting bullying, and ways of removing these barriers, are essential steps in the path forward. It is concerning that less than 50% of trainees in one study reported knowing who to contact when bullying occurs.^13^ Obstacles to reporting in a key study of bullying of UK NHS staff have been identified and include the belief that nothing would change, not wanting to be viewed as a troublemaker, the seniority of the perpetrator and uncertainty about how existing policies would be enacted and specific bullying allegations managed.^21^ Psychiatry trainees may fear punitive repercussions from senior colleagues or managers/administrators as well as not being taken seriously or being labelled as having a ‘victim mentality’. This would be consistent with the qualitative data in the Australian study describing that trainees reported being ridiculed and their grievances related to bullying being ‘laughed off’.^17^ The Australian study of surgical specialties, which included trainees, found that 44.7% of survey respondents indicated that they did not report bullying, so perhaps this may be common among Australian trainees.^24^

The finding in the West Midlands study that foreign trainees were significantly less likely to take action when bullied raises concerns.^13^ As the authors of that study point out, this may be due to the overseas trainees deciding that the incentives for challenging and confronting the bullying behaviours were outweighed by the gains from remaining quiet or colluding with the perpetrator. These trainees may be loath to ‘upset the apple cart’ and risk alienating consultants who they may be relying on for a reference or endorsement of their continued training and associated visa issues. They may also feel judged by their peers, who they fear may wrongly attribute the issues to ‘acculturation’ factors. Furthermore, being in a foreign country away from their usual social supports may also contribute to reluctance to confront perpetrators of bullying.

Awareness and prevention of bullying are also key steps in the path forward. As revealed in the Pakistan study, some trainees who reported experiencing bullying behaviours over the preceding 12 months in the Quine questionnaire did not report being bullied when asked directly in the survey.^16^ Even more concerning was the finding in the Australian study that several trainees viewed clearly bullying behaviours as a normal experience of training. Therefore, awareness programmes should be part of trainee orientation processes, with regular booster sessions over the course of training. In light of the finding in the West Midlands study that less than half (46%) of respondents reported that they did not know who to contact if bullied, there should be clear, easily accessible anti-bullying policies, protocols and codes of conduct.^13^ Consultant psychiatrists need to be aware of their responsibility in preventing workplace bullying and the range of behaviours that trainees may perceive as bullying.

Trainees need to be made aware of these resources in combination with active implementation by management. Given that many trainees seem reluctant to report and pursue assertive action against bullying, management needs to adopt proactive approach, including clearly communicated guidelines for trainees, perhaps during orientation, and with reinforcement each year of training, outlining the steps that need to be taken if bullying occurs. A ‘zero-tolerance’ policy on bullying by staff should be adopted by health services, medical boards and psychiatric colleges. A broad framework includes documentation of the bullying, reporting, and support from, and discussion with, peers and members of medico-political organisations such as medical associations, colleges of psychiatrists and unions.^32^

That the consequences of bullying can be devastating is highlighted by the previously cited study of the Australian surgical specialty, where 10.5–18.5% of those bullied left their jobs.^24^

Further research is therefore required with larger, more methodologically robust studies, exploring the nature, intensity, amount and contexts of bullying of psychiatric trainees. Perhaps data on bullying of psychiatric trainees are being collected by mandatory feedback processes for deaneries/local training schemes and directors of training but, owing to its sensitive nature and implications, the information is accessible to only a select few.

### Strengths and limitations

#### Strengths

The response rates in the five included studies were acceptable: all bar one above 70%, and 85% in one study. The review included studies of psychiatric trainees in the UK and abroad (Pakistan and Australia). Two studies used a wide sampling frame (all trainees registered with the Pakistan College of Physicians and Surgeons^16^) and a large UK psychiatric rotation.^14^ Two studies used a bullying assessment tool developed by Quine and based on an extensive literature review, even though not subjected to rigorous psychometric evaluation. The Australian study used a locally developed (‘purpose-built’) tool which was reviewed by senior research staff (and found to have face validity and comprehensiveness of content).^17^ Studies reported on how their surveys were administered. The Pakistan study obtained useful sociodemographic data as well as number of years of training.^16^ The analysis and reporting of results were generally adequate and all studies provided useful discussions of their results and the implications.

#### Limitations

There are many limitations to this review. It included only five studies informing on bullying of psychiatric trainees with primary data, and only one has been conducted within the past 10 years. The lack of recent studies is particularly important given the apparent shift that has occurred in the past 10–20 years regarding the (un)acceptability of bullying and sexual harassment within medical training, and in society more broadly. With the expansion of social media, and the MeToo movement (which originated in the context of sexual violence in the community), there may be increasing sharing of information on bullying, including how to deal with it. Furthermore, quality assessment indicated methodological weaknesses in the included studies (Table 2). The study designs in the papers were cross-sectional surveys with no comparator group (e.g. trainees in another faculty) and uncontrolled potential confounders, such as socioeconomic status and possible mental health problems of some trainees, which may make them more likely to report bullying (reporting bias). In some studies of bullying of junior doctors generally (not the studies in our review, which focused on psychiatric trainees), a mood dispositional dimension called negative affectivity has been controlled for, as people who are high in this item are more likely to report distress and grievances.^12,33^ However, this was not performed in the studies in our review. As cross-sectional surveys inviting voluntary participation from trainees, all studies in this review are subject to self-selection bias. Although two studies^13,15^ in our systematic review included ethnic background and overseas doctors training in the UK as part of the sociodemographic data collected, the remainder did not. In addition, studies would have been enhanced if more training variables and occupational health variables had been included. These would have enabled a better exploration and analysis of possible predictors of bullying and adjustment for potential confounders. All studies lacked the employment of an instrument with demonstrated psychometric reliability and validity, such as the NAQ-R. In addition, the inclusion of a measure of psychological distress such as the Kessler Psychological Distress Scale (K10) would have been useful. Only one study explored outcomes when bullied trainees sought help and support.^13^ Possible recall bias is another limitation of these cross-sectional surveys.

**Table 2:** Quality assessment of studies

Study	Selection bias	Study design	Confounders	Masking (‘blinding’)	Data collection method	Withdrawal and drop-out	Global rating
Ahmer et al (2009)^16^	Moderate	Weak	Weak	Weak	Moderate	Strong	Weak
Hoosen & Callaghan (2004)^13^	Moderate	Weak	Weak	Weak	Moderate	Strong	Weak
Reddy & Kaplan (2013)^15^	Moderate	Weak	Weak	Weak	Moderate	Strong	Weak
Morgan & Porter (1999)^14^	Moderate	Weak	Weak	Weak	Moderate	Strong	Weak
Kozlowska et al (1997)^17^	Strong	Weak	Weak	Weak	Moderate	Moderate	Weak

Quality assessment tool for quantitative studies (Effective Public Health Practice Project).

### Implications for further research

There are a surprisingly small number of studies investigating workplace bullying among psychiatric trainees, in the context of a medical specialty that provides mental healthcare and is therefore focused on holistic approaches to health and well-being, especially considering that consultant psychiatrists are often primarily responsible for the apprenticeship and creation of positive learning environments for trainees.

Although the reviewed studies have methodological limitations, the available evidence indicates that there is a substantial level of bullying of psychiatric trainees, and limited help-seeking by trainees for such bullying. Unfortunately, these findings are similar to those for the broader medical profession.

Further research is required with larger, more methodologically robust studies, exploring the nature, intensity, amount and contexts of bullying among psychiatric trainees. Perhaps data on bullying of psychiatric trainees are being collected by mandatory feedback processes for deaneries/local training schemes and directors of training but owing to its sensitive nature and implications, the information is accessible to only a select few. So, peer-reviewed published research is required to better ascertain the nature and extent of bullying across the profession, including in psychiatric specialist training, worldwide. Such research should include the use of standardised self-assessment surveys, based on agreed definitions of bullying, to allow for comparability of measurements, for example the Negative Acts Questionnaire or similar with local adaptations for language and context. However, to have real-world utility, any further studies should also usefully describe the psychiatric training programme and, at least briefly, the health system and sociocultural context in order to understand whether the findings are relevant to different contexts.

Currently, international healthcare workplace staffing shortages from the sequelae of the pandemic compound pre-existing workforce issues.^34^ In this context, understanding how bullying may be occurring in psychiatric training is necessary to prevent harm and hopefully stem bullying-mediated exits from the profession.

## About the authors

**Paul A. Maguire** is a senior lecturer and co-deputy head in the Academic Unit of Psychiatry and Addiction Medicine, The Australian National University School of Medicine and Psychology, Canberra Hospital, Canberra, Australia and a member of the Consortium of Australian Academic Psychiatrists for Independent Policy and Research Analysis (CAPIPRA), Canberra, Australia. **Fiona A. Wilkes** is a psychiatry advanced trainee in the Academic Unit of Psychiatry and Addiction Medicine, The Australian National University School of Medicine and Psychology, Canberra Hospital, Canberra, Australia and a member of CAPIPRA, Canberra, Australia. **Stephen Allison** is a member of CAPIPRA, Canberra, Australia and associate professor in the College of Medicine and Public Health, Flinders University, Adelaide, Australia. **Tarun Bastiampillai** is a member of CAPIPRA, Canberra, Australia, professor in the College of Medicine and Public Health, Flinders University, Adelaide, Australia and adjunct clinical professor in the Department of Psychiatry, Monash University, Melbourne, Australia. **Matt Brazel** is a clinical lecturer in the Academic Unit of Psychiatry and Addiction Medicine, The Australian National University School of Medicine and Psychology, Canberra Hospital, Canberra, Australia and a member of CAPIPRA, Canberra, Australia. **Jeffrey C. L. Looi** is associate professor and discipline lead in the Academic Unit of Psychiatry and Addiction Medicine, The Australian National University School of Medicine and Psychology, Canberra Hospital, Canberra, Australia and a member of CAPIPRA, Canberra, Australia.

## Supporting information

Maguire et al. supplementary material Maguire et al. supplementary material

## Data Availability

No new data were created in this study. The data cited can be found in the original articles listed in the references.

## References

[1] Quine L. Workplace bullying in NHS community trust: staff questionnaire survey. BMJ 1999; 318: 228–32. 9915730 10.1136/bmj.318.7178.228 PMC27703

[2] Psychiatrists’ Support Service. Bullying and Harassment (PSS Information Guide). Royal College of Psychiatrists, 2023 (https://www.rcpsych.ac.uk/docs/default-source/members/supporting-you/pss/pss-guide-6-bullying.pdf).

[3] Einarsen S, Hoel H, Notelaers G. Measuring exposure to bullying and harassment at work: validity, factor structure and psychometric properties of the negative acts questionnaire-revised. Work Stress 2009; 23: 24–44.

[4] Bartlett JE, Bartlett ME. Workplace bullying: an integrative literature review. Adv Dev Hum Resour 2011; 13: 69–84.

[5] Fair Work Ombudsman. *Bullying in the Workplace*. Australian Government, 2023 (https://www.fairwork.gov.au/employment-conditions/bullying-sexual-harassment-and-discrimination-at-work/bullying-in-the-workplace).

[6] Spector PE, Fox S. Theorizing about the deviant citizen: an attributional explanation of the interplay of organizational citizenship and counterproductive work behavior. Hum Resour Manag Rev 2010; 20: 132–43.

[7] Erwandi D, Kadir A, Lestari F. Identification of workplace bullying: reliability and validity of Indonesian version of the Negative Acts Questionnaire-Revised (NAQ-R). Int J Environ Res Public Health 2021; 18: 3985. 33920092 10.3390/ijerph18083985 PMC8070247

[8] Hoel H. Workplace bullying in United Kingdom. Workplace Bullying Harassment 2013; 12: 61–76.

[9] Cooper-Thomas H, Gardner D, O'Driscoll M, Catley B, Bentley T, Trenberth L. Neutralizing workplace bullying: the buffering effects of contextual factors. J Manag Psychol 2013; 28: 384–407.

[10] Scott J, Blanshard C, Child S. Workplace bullying of junior doctors: cross-sectional questionnaire survey. N Z Med J 2008; 121: 10–4. 18815599

[11] Askew DA, Schluter PJ, Dick M-L, Régo PM, Turner C, Wilkinson D. Bullying in the Australian medical workforce: cross-sectional data from an Australian e-cohort study. Aust Health Rev 2012; 36: 197–204. 22624642 10.1071/AH11048

[12] Quine L. Workplace bullying in junior doctors: questionnaire survey. BMJ 2002; 324: 878–9. 11950736 10.1136/bmj.324.7342.878 PMC101400

[13] Hoosen IA, Callaghan R. A survey of workplace bullying of psychiatric trainees in the West Midlands. Psychiatr Bull 2004; 28: 225–7.

[14] Morgan JF, Porter S. Sexual harassment of psychiatric trainees: experiences and attitudes. Postgrad Med J 1999; 75: 410–3. 10474725 10.1136/pgmj.75.885.410 PMC1741298

[15] Reddy S, Kaplan C. Abuse in the workplace: experience of specialist registrars. Psychiatr Bull 2006; 30: 379–81.

[16] Ahmer S, Yousafzai A-W, Siddiqi M, Faruqui R, Khan R, Zuberi S. Bullying of trainee psychiatrists in Pakistan: a cross-sectional questionnaire survey. Acad Psychiatry 2009; 33: 335–9. 19690120 10.1176/appi.ap.33.4.335

[17] Kozlowska K, Nunn K, Cousens P. Adverse experiences in psychiatric training. Part 2. Aust N Z J Psychiatry 1997; 31: 641–52. 9400870 10.3109/00048679709062676

[18] Coverdale JH, Balon R, Roberts LW. Mistreatment of trainees: verbal abuse and other bullying behaviors. Acad Psychiatry 2009; 33: 269–73. 19690101 10.1176/appi.ap.33.4.269

[19] Kozlowska K, Nunn K, Cousens P. Training in psychiatry: an examination of trainee perceptions. Part 1. Aust N Z J Psychiatry 1997; 31: 628–40. 9400869 10.3109/00048679709062675

[20] Colaprico C, Addari S, La Torre G. The effects of bullying on healthcare workers: an umbrella review of systematic reviews and meta-analyses. Rivista di Psichiatr 2023; 58: 41–9. 10.1708/4022.39973 37070330

[21] Carter M, Thompson N, Crampton P, Morrow G, Burford B, Gray C, et al. Workplace bullying in the UK NHS: a questionnaire and interview study on prevalence, impact and barriers to reporting. BMJ Open 2013; 3: e002628.

[22] Rashid S, Ullah A, Satti DI, Malik J, Iqbal H, Mehmoodi A, et al. Bullying in cardiology: Pakistan's perspective. Curr Probl Cardiol 2023; 48(7): 101691. 36921651 10.1016/j.cpcardiol.2023.101691

[23] Camm CF, Joshi A, Moore A, Sinclair HC, Westwood M, Greenwood JP, et al. Bullying in UK cardiology: a systemic problem requiring systemic solutions. Heart 2022; 108: 212–8. 34872975 10.1136/heartjnl-2021-319882

[24] Crebbin W, Campbell G, Hillis DA, Watters DA. Prevalence of bullying, discrimination and sexual harassment in surgery in Australasia. ANZ J Surg 2015; 85: 905–9. 26510837 10.1111/ans.13363

[25] Clements JM, King M, Nicholas R, Burdall O, Elsey E, Bucknall V, et al. Bullying and undermining behaviours in surgery: a qualitative study of surgical trainee experiences in the United Kingdom (UK) & republic of Ireland (ROI). Int J Surgery 2020; 84: 219–25. 10.1016/j.ijsu.2020.07.031 32738542

[26] Australian Health Practitioner Regulation Agency. *Medical Training Survey: Create Your Own Report*. AHPRA, 2023 (https://medicaltrainingsurvey.gov.au/results/create-your-own-report [accessed 14 Oct 2023)].

[27] Wilkes FA, Munindradasa A, Maguire PA, Anderson K, Looi JCL. Bullying within specialist medical training in Australia: analysis of the medical training survey, 2020–2023. Australasian Psychiatry [Epub ahead of print] 3 Aug 2024. Available from: 10.1177/10398562241269123. 39096080

[28] Steadman L, Quine L, Jack K, Felix DH, Waumsley J. Experience of workplace bullying behaviours in postgraduate hospital dentists: questionnaire survey. Br Dent J 2009; 207: 379–80. 19851385 10.1038/sj.bdj.2009.901

[29] Rayner C, Hoel H. A summary review of literature relating to workplace bullying. J Community Appl Soc Psychol 1997; 7: 181–91.

[30] Kivimäki M, Elovainio M, Vahtera J. Workplace bullying and sickness absence in hospital staff. Occup Environ Med 2000; 57: 656–60. 10984336 10.1136/oem.57.10.656 PMC1739873

[31] Kivimäki M, Virtanen M, Vartia M, Elovainio M, Vahtera J, Keltikangas-Järvinen L. Workplace bullying and the risk of cardiovascular disease and depression. Occup Environ Med 2003; 60: 779–83. 14504368 10.1136/oem.60.10.779 PMC1740404

[32] Looi JC, Allison S, Bastiampillai T. Reflections on, and responses to, managerial adverse reactions to healthcare advocacy by psychiatrists and trainees. Australas Psychiatry 2022; 30: 158–61. 34814761 10.1177/10398562211040463

[33] Quine L. Workplace bullying, psychological distress, and job satisfaction in junior doctors. Camb Q Healthc Ethics 2003; 12: 91–101. 12625206 10.1017/s0963180103121111

[34] Looi JC, Allison S, Bastiampillai T, Kisely SR, Robson SJ. Supply and demand – a health economic perspective on the Australian hospital and elective surgery crisis. Aust Health Rev 2023; 47: 391–3. 37339737 10.1071/AH23048

