# Supplementary material for: Workplace bullying of psychiatric trainees: systematic review
Source: BJPsych Bull. 2025 Oct;49(5):315–24. doi: 10.1192/bjb.2024.58 (PMC12501511; doi:10.1192/bjb.2024.58)
Supplement: Maguire et al. supplementary material [file S2056469424000585sup001.docx]

**Appendix**

**Search strategy for Ovid MEDLINE, CINAHL, PsychINFO, and EMBASE:**1 Bullying.mp. or *sexual bullying/ or exp bullying/

2 Harassment.mp or exp harassment/

3 Intimidation.mp or exp intimidation/

4 Discrimination.mp

5 Workplace abuse.mp

6 Abuse in the workplace.mp

7 Psychiatr* trainee*.mp
8 Psychiatr* registrar*.mp

9 Psychiatr* resident*.mp

10  Psychiatr* intern*.mp

11  Specialist registrar*.mp

12  Trainee psychiatrist*.mp

13  1 or 2 or 3 or 4 or 5 or 6

14  7 or 8 or 9 or 10 or 11 or 12

15  13 and 14

**Search strategy for PubMed**

(Bullying OR harassment OR intimidation OR discrimination OR workplace abuse OR abuse in the workplace) AND (psychiatr* trainee* OR psychiatr* registrar* OR psychiatr* resident* OR psychiatr* intern* OR specialist registrar* OR trainee psychiatrist*)

*represents alternative (e.g. plural) forms of the relevant word
